# The potential to control *Haemonchus contortus* in indigenous South African goats with copper oxide wire particles

**DOI:** 10.1016/j.vetpar.2009.03.005

**Published:** 2009-06-10

**Authors:** A.F. Vatta, P.J. Waller, J.B. Githiori, G.F. Medley

**Affiliations:** a Onderstepoort Veterinary Institute, Private Bag X05, Onderstepoort 0110, South Africa; b National Veterinary Institute, Department of Parasitology (SWEPAR), Uppsala SE-751 89, Sweden; c International Livestock Research Institute, P.O. Box 30709, Nairobi 00100, Kenya; d University of Warwick, Department of Biological Sciences, Coventry CV4 7AL, United Kingdom

**Keywords:** Anthelmintic resistance, Copper oxide, COWP, Goat, *Haemonchus contortus*

## Abstract

The high prevalence of resistance of *Haemonchus contortus* to all major anthelmintic groups has prompted investigations into alternative control methods in South Africa, including the use of copper oxide wire particle (COWP) boluses. To assess the efficacy of COWP against *H. contortus* in indigenous South African goats, 18 male faecal egg-count-negative goats were each given *ca.*1200 infective larvae of *H. contortus* three times per week during weeks 1 and 2 of the experiment. These animals made up an “established” infection group (ESTGRP). At the start of week 7, six goats were each given a 2-g COWP bolus orally; six goats received a 4-g COWP bolus each and six animals were not treated. A further 20 goats constituted a “developing” infection group (DEVGRP). At the beginning of week 1, seven of the DEVGRP goats were given a 2-g COWP bolus each; seven goats were treated with a 4-g COWP bolus each and no bolus was given to a further six animals. During weeks 1–6, each of these DEVGRP goats was given *ca.* 400 *H. contortus* larvae three times per week. All 38 goats were euthanized for worm recovery from the abomasa and small intestines in week 11. In the ESTGRP, the 2-g and 4-g COWP boluses reduced the worm burdens by 95% and 93%, respectively compared to controls (mean burden ± standard deviation, SD: 23 ± 33, 30 ± 56 and 442 ± 518 worms, *P* = 0.02). However, in the DEVGRP goats, both the 2-g and 4-g COWP treatments were ineffective in reducing the worm burdens relative to the controls (mean burdens ± SD: 1102 ± 841, 649 ± 855, 1051 ± 661 worms, *P* = 0.16). Mean liver copper levels did not differ between the ESTGRP goats treated with 2-g COWP, 4-g COWP or no COWP (mean ± standard error of the mean, SEM, in ppm: 93.7 ± 8.3; 101.5 ± 8.3; 71.8 ± 8.3, *P* = 0.07) nor did they differ between the DEVGRP goats (mean ± SEM, in ppm: 74.1 ± 9.1; 75.4 ± 9.1; 74.9 ± 10.0, *P* > 0.99). The copper values were considered adequate, but not high, for goats. The COWP boluses have the potential to be used in the place of conventional anthelmintics for the control of established *H. contortus* infections in indigenous South African goats, but their use as part of an integrated approach to control *H. contortus* in the field must be fully investigated.

## 1 Introduction

*Haemonchus contortus* is a major constraint to the production of sheep and goats in the tropical and subtropical regions of the world and causes substantial economic losses to small-scale farmers (Perry et al., 2002). Control of the parasite is complicated by the presence of widespread resistance to the available anthelmintic groups (Vatta and Lindberg, 2006). This has prompted investigations into alternative methods of control (Waller, 2006; Torres-Acosta and Hoste, 2008), of which treatment with copper oxide wire particles (COWP) is attracting considerable research interest (Burke et al., 2007a,b).

Developed initially to overcome copper deficiency in grazing ruminants (Dewey, 1977; Suttle, 1981), the COWP-containing bolus releases particles in the rumen. These then move distally through the gut of the animals to lodge in the mucosal folds of the abomasum. Here they dissolve to release ionic copper over several weeks which is absorbed from the intestinal tract and stored in the liver (Langlands et al., 1989). An additional benefit of COWP treatment is that it has been shown to reduce established burdens of *H. contortus* in infected sheep (Waller et al., 2004; Burke et al., 2004) and goats (Chartier et al., 2000). Several workers have also examined the prophylactic effect of COWP in preventing the establishment of infective larvae of *H. contortus*, but the efficacy of the treatments has varied (Knox, 2002; Chartier et al., 2000; Martínez Ortiz de Montellano et al., 2007).

Although COWP is gaining credence as an anthelmintic therapy, there are a number of issues that have yet to be addressed. Because differences in the metabolism of copper have been observed between sheep and goat breeds (Rankins et al., 2002), the aims of the present study were to assess whether COWP boluses have an anthelmintic effect against established and establishing infections of *H. contortus* in indigenous goats of South Africa. For animals that are not copper deficient, there is a concern that supplementation with COWP will cause copper toxicity and that it may affect meat quality. A further aim of the present study was thus to assess the levels of copper in the tissues of the goats at slaughter, following treatment with COWP.

## 2 Materials and methods

### 2.1 Source of goats and their management

The work described in this article was reviewed and approved by the Onderstepoort Veterinary Institute Animal Ethics Committee (Project OV 21/10/C131). Forty-four 100–120-day-old indigenous castrated male Zulu goats were purchased from an agricultural experimental station near Pietermaritzburg, South Africa and transported to the Onderstepoort Veterinary Institute, Pretoria, South Africa. Approximately 2 weeks prior to transport to the Institute, the goats were dewormed on-farm with 10 mg/kg fenbendazole (Panacur BS, Intervet South Africa) daily for 5 days (days −103 to −99, Tables 1 and 2). They were also given 12 mg/kg levamisole (Nemasol NF, Intervet South Africa) on the first day of fenbendazole treatment (day −103). However, within 6 days of arrival at the Onderstepoort Veterinary Institute, five of the goats died or were euthanized as they were showing severe signs of anaemia. Total worm counts for these animals indicated heavy *H. contortus* burdens (2005–9367 worms) with negligible counts for *Trichostrongylus* spp. (0–20 worms) and *Strongyloides papillosus* (0–10 worms).

The surviving goats were divided into two groups and maintained as an “established” infection group (ESTGRP) of 19 goats and a “developing” infection group (DEVGRP) of 20 goats (see Section 2.2). The DEVGRP animals were further dewormed with 80 mg/kg trichlorfon (Uni-Dose, Intervet South Africa) and 0.2 mg/kg ivermectin (Ivomec Liquid, Merial South Africa) *ca.* 24 h and 36 h following the trichlorfon treatment (Table 2). The ESTGRP goats were initially not given further treatment as it seemed appropriate to allow these existing infections to mature and then to test the efficacy of the copper oxide wire particles against them. However, substantial drops in the haematocrits of these animals also necessitated their treatment with the trichlorfon and ivermectin combination, 1 week after the DEVGRP goats (Table 1). In both groups, the trichlorfon and ivermectin treatment was at most 95% effective in reducing the egg counts. Subsequent treatment of all the goats with 0.4 mg/kg moxidectin (Cydectin Injectable, Fort Dodge Animal Health) proved effective in reducing the egg counts to zero in all animals but one. This animal had an egg count of 100 eggs per gram of faeces (epg), but was negative on egg count before the experiment proper was started.

The goats were kept in concrete pens that were swept clean of faeces on a daily basis and the animals were fed a commercial pelleted feed and kikuyu (*Pennisetum clandestinum*) hay. During week 2 of the experiment, one of the ESTGRP goats was euthanized while suffering from urolithiasis. Diets with a low calcium-to-phosphorus ratio are recognised to predispose to the development of phosphatic urinary calculi (Bath and De Wet, 2000). Because the calcium-to-phosphorus ratio of kikuyu is particularly low (Oosthuizen, 1994), the kikuyu hay was replaced with lucerne hay (*Medicago sativa*) which has a high calcium-to-phosphorus ratio (Shakespeare, 1996). The kikuyu hay was substituted with lucerne hay (50:50) in week 2 and totally replaced with lucerne hay during week 3 for both ESTGRP and DEVGRP goats. No further problems with urolithiasis were experienced after this change in diet. All the data for the animal that was euthanized were excluded from this article.

### 2.2 Experimental design

The goats were maintained in their pens for a further 10 weeks following moxidectin treatment before being allocated to the following treatments.

#### 2.2.1 Established infection group (ESTGRP, Table 3)

The goats of this group were each given *ca.* 1200 3rd-stage *H. contortus* larvae three times per week for 2 weeks (weeks 1 and 2). Faecal egg counts were carried out 5 weeks after the start of the artificial infection (week 6) and the goats were ranked in order of descending egg count. The three goats with the highest egg counts were randomly allocated to one of three experimental groups. The next three goats were then randomly allocated to the three treatment groups. This process was repeated until all 18 goats had been allocated to a treatment group. Six weeks after the start of the artificial infection (week 7), six goats were treated with a 2-g COWP bolus (Copinox Lamb, Animax Ltd., UK) each, six goats received a 4-g COWP bolus (Copinox Ewe/Calf, Animax Ltd., UK) each and six animals were left as untreated controls. All the goats were euthanized for worm recovery from the abomasum and small intestine 4 weeks later (week 11). Samples of liver, kidney and muscle were taken at slaughter and analysed for copper levels. The goats were weighed during weeks −2 and 10.

#### 2.2.2 Developing infection group (DEVGRP, Table 4)

The goats of this group were divided into three groups of six to seven animals each. The groups were balanced for liveweight in a manner described by Vatta et al. (2008). To do this, the goats were weighed during week −2 and were ranked in order of descending liveweight. The three goats with the highest liveweights were randomly allocated to one of the treatment groups. Then the next three heaviest goats were randomly assigned to one of the three groups. This process was followed until all 20 goats had been allocated to treatment groups. During week 1, one group of seven goats received a 2-g COWP bolus each; seven goats received a 4-g COWP bolus each and six animals were left as untreated controls. The goats were each artificially infected with *ca.* 400 3rd-stage larvae of *H. contortus* three times per week for 6 weeks (weeks 1–6), starting on the day of treatment with COWP. The goats were euthanized for worm recovery 10 weeks after the first larvae had been administered (week 11). Copper levels in the liver, kidney and muscle were determined at slaughter. The goats were again weighed in the week prior to slaughter (week 10).

### 2.3 Infection of goats

The preparation and administration of larval inocula followed the guidelines of Wood et al. (1995). Two worm-free donor sheep were infected with a susceptible strain of *H. contortus* and 3rd-stage larvae derived from the culture of faeces of these donor sheep were used to infect the experimental goats. These larvae were stored in tap water in flat-sided glass bottles at approximately 10 °C until use and were generally 7–11 days old when the goats were infected. The larvae were administered orally to each goat by depositing the correct dose of larvae over the base of the tongue using a plastic syringe.

### 2.4 Parasitological methods

The faecal egg counts and faecal cultures were carried out according to Reinecke (1983), while the key of Van Wyk et al. (2004) was used for the identification of the 3rd-stage nematode larvae.

Worm burdens of the abomasum and small intestine of each animal were estimated at slaughter according to the methods of Wood et al. (1995). The nematodes in two 10% aliquots of the contents of each organ were recovered and counted. The first 15 nematodes to be counted per aliquot were mounted on microscope slides and identified according to Visser et al. (1987). The mucosae of the abomasum and small intestine were subjected to peptic digestion (Ministry of Agriculture, Fisheries and Food, 1986) and all the nematodes in the digested material were recovered and counted and again the first 15 nematodes to be counted were identified. The average count for the two aliquots of each organ was determined, multiplied by 10 and this number added to the count for the digested material to give the total number of nematodes for that organ. The percentage reduction in treated groups relative to controls was calculated according to the formula, ((*C* − *T*)/*C*) × 100, where *C* and *T* are the arithmetic means of the untreated and treated groups, respectively.

The liver, kidney and muscle samples were analysed for copper on a wet matter basis according to the method of Boyazoglu et al. (1972). This method employed an acid digestion technique and values were determined on an atomic absorption spectrophotometer (GBC 908 AA, GBC Scientific Equipment, Dandenong, Australia).

### 2.5 Statistical analysis

The data were analysed statistically by means of an analysis of variance (ANOVA) for unbalanced data using the GenStat statistical software (GenStat^®^ for Windows^®^ 7th edition, VSN International Ltd.). The effects of COWP treatment on faecal egg count, total worm count, tissue copper levels, liveweight and liveweight gain were examined. The treatment means were separated using Fisher's protected *t*-test least significant difference at the 5% level. The numbers of male and female *H. contortus* recovered were compared overall and within the ESTGRP and DEVGRP goats by means of a two-sample *t*-test. The data for the faecal egg counts and worm counts were log-transformed [*y* = log_10_ (*x* + 1)] prior to analysis to stabilize treatment variances, but the untransformed values are reported in this article.

## 3 Results

With the exception of one *Nematodirus* spp. female recovered from one of the ESTGRP controls, all nematodes recovered were identified as *H. contortus*. The worm counts at slaughter were generally low (Tables 3 and 4). Approximately 7200 larvae were administered per goat but the percentage establishment per group was approximately 6% for the ESTGRP controls and 15% for the DEVGRP controls. The maximum number of worms found in any goat was 2480 worms for one of the DEVGRP 2-g COWP-treated goats and 1448 worms for one of the ESTGRP control goats.

Most of the worms recovered had developed to the adult stage. There were no statistically significant differences between the numbers of male and female *H. contortus* recovered from the goats overall in the ESTGRP and DEVGRP goats or between treatments within these groups (*P* > 0.05).

Within the ESTGRP goats, the number of *H. contortus* recovered in the COWP-treated groups at 2 g and 4 g dosages was respectively, 95% and 93% less than the control animals and the mean worm counts for the COWP-treated groups were significantly lower than the mean count for the controls (Table 3, *P* < 0.05).

Within the DEVGRP goats, COWP treatment at both dosages did not result in a statistically significant reduction in *H. contortus* recovery (Table 4), although the average number of worms recovered per goat from the group treated with 4 g COWP was lower than the averages for the other two groups.

Tissue copper levels in COWP-treated animals did not differ significantly from those in control animals. While the copper levels for liver and kidney in the treated ESTGRP goats were higher than those of the controls, the levels were within the range described as ‘adequate’ for goats (liver: 25–150 ppm wet weight and kidney: 3–6 ppm wet weight), i.e. neither marginal nor high (Puls, 1994). The copper levels in the muscle tissue were similar to those of the controls.

There were no differences in liveweight or liveweight gain in the ESTGRP goats at the end of the experiment (Table 3). However, the liveweights of the 4-g COWP group were significantly higher than the controls in the DEVGRP goats at the end of the experiment and the 4-g COWP-treated goats gained significantly more liveweight than the controls (Table 4).

## 4 Discussion

At both 2 g and 4 g doses COWP was effective in reducing artificially established worm burdens by 93–95% in indigenous South African goats. These results concur with those of other workers. Using a similar experimental design to the present experiment, Waller et al. (2004) found that treatment with a 4-g COWP bolus was 97% and 56% effective, respectively, in reducing 6-week-old burdens of adult and early 4th-stage larvae of *H. contortus* in sheep. Burke et al. (2004) demonstrated reductions of 90%, 94% and 93% of 4-week-old *H. contortus* infections in lambs, with doses of 2 g, 4 g and 6 g COWP, respectively. Chartier et al. (2000) obtained a 75% reduction in 4-week-old *H. contortus* burdens following administration of COWP at doses of 2–4 g to dairy goats.

In the present study, prophylactic treatment with COWP at both 2 g and 4 g dosages was ineffective in preventing the establishment of *H. contortus* larvae administered over a 6-week period as a trickle infection. Using the same experimental design as the present work, Waller et al. (2004) found that a dose of 4 g COWP was ineffective in preventing the development of *H. contortus* larvae administered to sheep over a 6-week period. However, Knox (2002) found a significant reduction in *H. contortus* burdens of 37% in a sample of the experimental sheep slaughtered at week 4 after the start of weekly infection with *H. contortus* larvae. In a second experiment described in this paper (Knox, 2002), the author found reductions of 33% (non-significant) and 54% (significant) in a proportion of the experimental sheep slaughtered at weeks 4 and 6, respectively. In both experiments, larvae were administered starting 5–7 days after 2.5 g COWP had been given per sheep. When Chartier et al. (2000) administered a 4-g COWP bolus to dairy goats, followed by artificial infection with *H. contortus* larvae for 8 weeks starting 1 week after the COWP had been given, the percentage reduction in worm burdens at the end of 8 weeks was 39% (non-significant). Martínez Ortiz de Montellano et al. (2007) administered two boluses of 2 g COWP at a 60-day interval to goat kids browsing and grazing on natural vegetation and found a non-significant reduction of 35% in *H. contortus* burdens when the kids were slaughtered 94 days after the second bolus had been given. The 38% reduction in worm burdens in the 4-g COWP-treated DEVGRP goats of the present study is thus in agreement with the results of these other studies. These goats also showed greater liveweight gains over the controls. This effect may have been a result of the partial removal of the worms by the COWP treatment. On the other hand, Bang et al. (1990a) administered a dosage of 5 g COWP per sheep 5 days before the sheep were artificially infected with *H. contortus* larvae on three occasions separated by 3 days. At slaughter 21–22 days after the last infection, the reduction of worm burdens in the treated sheep relative to the controls was 96%. Since the infective larvae were given over a period of 7 days, this would mean that the COWP had been effective in preventing incoming larvae from establishing themselves over a 12-day period.

Several authors have recovered copper particles from the abomasum of sheep at slaughter, which occurred from 23 to 44 days after treatment with COWP (Langlands et al., 1989; Bang et al., 1990a,b). However, the anthelmintic effect of COWP is thought to be related to the concentration of ionic copper liberated from the COWP in the abomasum (Bang et al., 1990a) and the level required to kill the nematodes appears to be maintained for a shorter duration. Bang et al. (1990b) demonstrated that proximal duodenal concentrations of soluble copper increased gradually for 4 days following administration of COWP, stabilized at *ca.* 0.1 μg/g digesta in animals infected with *Teladorsagia circumcincta* and at 0.25 μg/g digesta in uninfected animals for 10 days, and then declined. In another study, Langlands et al. (1989) found that mean daily faecal excretion of copper by sheep given COWP increased to peak levels on days 5 and 6 and declined thereafter. These differences were significant from day 3 to days 13–14 and again on days 17–18. It seems therefore that the levels of soluble copper in the abomasum are only sufficiently raised to kill abomasal nematodes for at most 2 weeks after administration of the COWP. The exact concentration of soluble copper required for the anthelmintic effect to be seen has not yet been determined. It might be possible that the delivery of ionic copper could be optimised for a longer duration of effect. However, it should be noted that this will induce a selective pressure for *H. contortus*, so that genetic variants that can survive high copper concentrations will be favoured.

Goats appear to be less susceptible to copper toxicity than sheep and there is thus a lesser chance of copper toxicity arising from the use of COWP in goats than in sheep (Navarre and Pugh, 2002). However, sheep and goat breeds may metabolise copper differently (Osman et al., 2003; Rankins et al., 2002). A few studies have examined copper levels in relation to the anthelmintic use of copper oxide. In Katahdin, Dorper or Dorper crossbred lambs given 2 g and 4 g COWP and slaughtered 4–5 weeks after treatment, Burke et al. (2004) reported levels of 136 ppm and 161 ppm wet weight compared with a level of 62 ppm wet weight for the controls. Values of 100–500 ppm wet weight are considered high in sheep (Puls, 1994). Burke et al. (2005) also measured AST activity, an indicator of liver copper levels, in Katahdin lambs whose dams had been given COWP approximately 1 month prior to lambing. They found that the AST activity at 30 days after birth was higher in the lambs of those dams given 4 g COWP than in the lambs of those dams given 0 g or 2 g COWP. In Polypay ewes given 0 g, 0.5 g, 0.75 g, 1 g or 2 g COWP 2 months after lambing and their lambs given 0 g, 0.5 g, 0.75 g, 1 g or 2 g COWP two-and-a-half months after lambing, the AST activity was similar among the groups at 28 days after treatment (Burke et al., 2007a). These results suggest that there were no differences in copper metabolism between the Katahdin and Polypay sheep breeds when the COWP was given at a dose of 2 g or less. In Spanish-Boer crossbred goats given 0 g, 5 g or 10 g COWP and slaughtered 35 days after treatment, Burke et al. (2007b) found that while the concentrations were greater in the treated groups (5 g: 153 mg/kg of dry matter; 10 g: 149 mg/kg of dry matter) than in the controls (56 mg/kg of dry matter), the values were in the lower range of the values considered adequate for goats (Puls, 1988, cited by Burke et al., 2007b). Similarly, in the indigenous South African goats in the present study, the levels of copper in the liver and kidney of the treated goats at slaughter were also considered adequate, suggesting that breed difference did not play a significant role in the metabolism of the copper. The concentrations of copper in the muscle tissue were not significantly different from those of the untreated controls in the present study, indicating that the meat would be suitable for human consumption provided a 4-week period has elapsed following a single treatment. However, the frequency with which COWP may safely be used in goats under South African farming and agro-ecological conditions still needs to be evaluated. Copper therapy should not be applied in areas where accumulation of copper in plants is known to occur, such as the central Western Cape, western Eastern Cape and southern Free State Provinces (Bath, 1979).

Van Wyk et al. (1989) reported that ivermectin, closantel and disophenol had a geometric mean efficacy of 94.8% or more in a population of *H. contortus* isolated from sheep in June 1987 from the same agricultural experimental station from which the goats for this study were purchased. However, they reported resistance to levamisole, morantel, rafoxanide and oxfendazole. At that time, therefore, drugs from three anthelmintic groups had decreased substantially in their claimed efficacy against the parasite. The present investigation, conducted 19 years later, indicates a situation that has worsened. The *H. contortus* was still resistant to levamisole and the benzimidazoles, but a combination of trichlorfon and ivermectin was also not completely effective in reducing the faecal egg counts in the goats. The efficacy of the combination treatment may have been as low as 50% or 68%, although the calculation of the latter two efficacies for the DEVGRP goats was based on egg counts taken 2 days prior to the actual treatment and there may have been some shedding of worms during these 2 days. However, at most, the combination was only 95% effective as measured in the ESTGRP goats. Only off-label use of moxidectin in goats at double the recommended dosage for sheep can at present probably be safely relied upon to have an adequate effect, but, as Kaplan et al. (2007) have discussed, the presence of resistance to ivermectin may be indicative of the development of resistance to moxidectin.

The management of the goats on the agricultural experimental station from which the resistant *H. contortus* was isolated was not supportive of sustainable parasite control. The average annual rainfall of 800–850 mm which is received mainly in the summer (Van Wyk et al., 1989) is conducive to the establishment of warm, humid conditions and to a thriving population of *H. contortus* on pasture. At the time that the goats were purchased, most of the goats on the farm were being maintained under conditions of lush planted pastures including kikuyu grass (*P. clandestinum*). The goats did not have access to browse and were thus forced to graze and, as such, had no chance of avoiding or reducing infection with *H. contortus* larvae. Changes of management would be necessary to include the better utilisation of natural pasture on the farm and a reduction in the number of goats to reduce the intensity of grazing. Instead of corralling the goats in small grass paddocks overnight, as was the practice, it would be useful to keep them overnight in raised houses with slatted floors, to allow the faeces to fall through on to the floor beneath the houses where they could be removed once a week. The use of COWP may provide an alternative to the conventional anthelmintics for this farm, but its efficacy against the resistant *H. contortus* population on the farm still needs to be evaluated. Nevertheless, the future use of COWP would have to be judicious if its efficacy were to be maintained. The inevitable rise of anthelmintic resistance suggests that future control of nematode infections will require an integrated, multi-faceted approach, and not rely on a relatively small number of chemotherapeutics. We believe that these results suggest that COWP can be a useful part of such an approach.

## Figures and Tables

**Table 1:** The anthelmintic treatment regimen employed in rendering the goats of the “established” infection group (ESTGRP) nematode-free following their purchase from an agricultural experimental farm near Pietermaritzburg, South Africa.

Day	Mean FEC in epg^a^ (range)	PR^b^	*n*
−103^c^	2225 (300–7500)	–	12
−103 to −99	Treatment A^d^		
−88	Moved off pasture to cement pens		
−84	2292 (700–3800)	−80% (days −103 to −84)	12
−75	2800 (100–5400)	–	12
−75 to −73	Treatment B^e^		
−69	150 (0–800)	95% (days −75 to −69)	12
−69	Treatment C^f^		
−56	0 (0–0)	100% (days −69 to −56)	12^g^

a Mean faecal egg count in eggs per gram of faeces.

b PR: mean percentage reduction where PR = (FEC_1_ − FEC_2_)/FEC_1_ × 100 where 1 represents the FEC pre-treatment and 2 the FEC post-treatment (Van Wyk and Van Wijk, 1992). The reduction per individual animal was first calculated before the mean for the group was determined (Cabaret and Berrag, 2004). Only data for goats for which egg counts were available on each date were included in the calculations.

c 25 January 2006 (15 weeks prior to the start of the experiment proper). Day 0 is the 1st day of week 1.

d 10 mg/kg fenbendazole (Panacur BS, Intervet South Africa) and 12 mg/kg levamisole (Nemasol NF, Intervet South Africa) on day −103 and 10 mg/kg fenbendazole on days −102 to −99.

e 80 mg/kg trichlorfon (Uni-Dose, Intervet South Africa); followed by 0.2 mg/kg ivermectin (Ivomec Liquid, Merial South Africa) *ca.* 24 h later and *ca.* 12 h after that.

f 0.4 mg/kg moxidectin (Cydectin Injectable, Fort Dodge Animal Health).

g *n* = 6 for the calculation of the PR. Only data for goats for which egg counts were greater than zero for the pre-treatment date were included in this last calculation.

**Table 2:** The anthelmintic treatment regimen employed in rendering the goats of the “developing” infection group (DEVGRP) nematode-free following their purchase from an agricultural experimental farm near Pietermaritzburg, South Africa.

Day	Mean FEC in epg^a^ (range)	PR^b^	*n*
−103^c^	4594 (400–11300)	–	17
−103 to −99	Treatment A^d^		
−88	Moved off pasture to cement pens		
−84	4053 (1300–8100)	−111% (days −103 to −84)	17
−82 to −81	Treatment B^e^		
−75	859 (0–4400)	68% (days −84 to −75)	17
−69	1182 (0–4400)	50% (days −84 to −69)	17
−69	Treatment C^f^		
−56	6 (0–100)	100% (days −69 to −56)	17^g^

a Mean faecal egg count in eggs per gram of faeces.

b PR: mean percentage reduction calculated as described for Table 1.

c 25 January 2006 (15 weeks prior to the start of the experiment proper). Day 0 is the 1st day of week 1.

d 10 mg/kg fenbendazole (Panacur BS, Intervet South Africa) and 12 mg/kg levamisole (Nemasol NF, Intervet South Africa) on day −103 and 10 mg/kg fenbendazole on days −102 to −99.

e 80 mg/kg trichlorfon (Uni-Dose, Intervet South Africa); followed by 0.2 mg/kg ivermectin (Ivomec Liquid, Merial South Africa) *ca.* 24 h later and *ca.* 12 h after that.

f 0.4 mg/kg moxidectin (Cydectin Injectable, Fort Dodge Animal Health).

g *n* = 15 for the calculation of the PR. Only data for goats for which egg counts were greater than zero for the pre-treatment date were included in this last calculation.

**Table 3:** Details of the infection with 3rd-stage larvae of *Haemonchus contortus* and treatment with copper oxide wire particle (COWP) boluses, for the established infection group (ESTGRP). COWP boluses were tested after the infection had become established.

Week		2-g group (*n* = 6)	4-g group (*n* = 6)	Control (*n* = 6)	*P*
−2	Mean liveweight ± SEM^a^ (kg)	18.6 ± 0.7	17.2 ± 0.7	18.1 ± 0.7	0.43
−2	Mean FEC ± SD (epg)^b^	0 ± 0	0 ± 0	0 ± 0	
1–2	Infection	1200 *H. contortus* larvae per goat, three times/week			
6	Mean FEC ± SD (epg)	1367 ± 2094	1483 ± 2273	1000 ± 967	0.48
7	Treatment	2 g COWP^c^	4 g COWP	Not treated	
10	Mean liveweight ± SEM (kg)	24.8 ± 0.9	22.4 ± 0.9	24.3 ± 0.9	0.19
10	Mean liveweight gain ± SEM (kg)	6.2 ± 0.4	5.2 ± 0.4	6.3 ± 0.4	0.17
11	Goats euthanized				
11	Mean FEC ± SD (epg)	150 ± 321	133 ± 197	1267 ± 2281	0.18
11	Mean total *H. contortus* ± SD (% 4th-stage larvae)	23 ± 33^†^ (0)	30 ± 56^†^ (21)	442 ± 518* (0)	0.02^d^
11	Mean liver [Cu]^e^ ± SEM (ppm)	93.7 ± 8.3	101.5 ± 8.3	71.8 ± 8.3	0.07
11	Mean kidney [Cu] ± SEM (ppm)	3.8 ± 0.6	4.7 ± 0.6	3.3 ± 0.6	0.33
11	Mean muscle [Cu] ± SEM (ppm)	1.3 ± 0.2	1.7 ± 0.2	1.3 ± 0.2	0.56

a Standard error of the mean.

b Faecal egg count ± standard deviation in eggs per gram of faeces (epg).

c Copper oxide wire particles (2 g: Copinox Lamb and 4 g: Copinox Ewe/Calf, Animax Ltd., UK).

d Means with different superscripts (^†,^*) differ significantly.

e Copper concentration in parts per million on a wet basis.

**Table 4:** Details of the infection with 3rd-stage larvae of *Haemonchus contortus* and treatment with copper oxide wire particle (COWP) boluses, for the developing infection group (DEVGRP). COWP boluses were given prophylactically before infection with larvae.

Week		2-g group (*n* = 7)	4-g group (*n* = 7)	Control (*n* = 6)	*P*
−2	Mean liveweight ± SEM^a^ (kg)	16.6 ± 0.2	17.0 ± 0.2	17.0 ± 0.3	0.52
−2	Mean FEC ± SD (epg)^b^	0 ± 0	0 ± 0	0 ± 0	
1	Treatment	2 g COWP^c^	4 g COWP	Not treated	
1–6	Infection	400 *H. contortus* larvae per goat, three times/week			
6	Mean FEC ± SD (epg)	214 ± 157	214 ± 324	750 ± 1198	0.69
10	Mean liveweight ± SEM (kg)	21.2 ± 0.5^†^	23.6 ± 0.5*	22.2 ± 0.5^†,^*	0.02^d^
10	Mean liveweight gain ± SEM (kg)	4.6 ± 0.3^†^	6.6 ± 0.3*	5.2 ± 0.3^†^	<0.01
11	Goats euthanized				
11	Mean FEC ± SD (epg)	3000 ± 2165	1629 ± 2458	1600 ± 1033	0.10
11	Mean total *H. contortus* ± SD (% 4th-stage larvae)	1102 ± 841 (1)	649 ± 855 (0)	1051 ± 661 (0)	0.16
11	Mean liver [Cu]^e^ ± SEM (ppm)	74.1 ± 9.1	75.4 ± 9.1	74.9 ± 10.0	>0.99
11	Mean kidney [Cu] ± SEM (ppm)	3.7 ± 0.6	3.5 ± 0.6	4.4 ± 0.6	0.52
11	Mean muscle [Cu] ± SEM (ppm)	1.3 ± 0.4	1.0 ± 0.4	2.0 ± 0.4	0.22

a Standard error of the mean.

b Faecal egg count ± standard deviation in eggs per gram of faeces (epg).

c Copper oxide wire particles (2 g: Copinox Lamb and 4 g: Copinox Ewe/Calf, Animax Ltd., UK).

d Means with different superscripts (^†,^*) differ significantly.

e Copper concentration in parts per million on a wet basis.
